# Diffusion Tensor Imaging of Fetuses With Congenital Diaphragmatic Hernia

**DOI:** 10.1002/pd.6835

**Published:** 2025-06-11

**Authors:** Usha D. Nagaraj, Jonathan A. Dudley, Kristin Lam, Beth M. Kline‐Fath, Stephanie L. Merhar, Francesco T. Mangano, Weihong Yuan

**Affiliations:** ^1^ Department of Radiology and Medical Imaging Cincinnati Children's Hospital Medical Center Cincinnati Ohio USA; ^2^ University of Cincinnati College of Medicine Cincinnati Ohio USA; ^3^ Cincinnati Children's Hospital—Imaging Research Center Cincinnati Ohio USA; ^4^ Department of Pediatrics Cincinnati Children's Hospital Medical Center Cincinnati Ohio USA; ^5^ Division of Neonatology Cincinnati Children's Hospital Medical Center Perinatal Institute Cincinnati Ohio USA; ^6^ Department of Neurosurgery Cincinnati Children's Hospital Medical Center Cincinnati Ohio USA

**Keywords:** congenital diaphragmatic hernia, diffusion tensor imaging, fetal MRI

## Abstract

**Objective:**

To evaluate differences in diffusion tensor imaging (DTI) parameters in the brain between fetuses with congenital diaphragmatic hernia (CDH) and age‐matched controls.

**Method:**

This retrospective IRB‐approved study included fetal MRIs for CDH and gestational age (GA) matched controls with lung pathology other than CDH with normal fetal brains. Fetal DTI data were acquired in 15 noncollinear diffusion‐weighting directions with the b‐value at 600 s/mm^2^ (1 b0). Slice‐to‐volume registration (SVR) was employed to correct for motion artifact.

**Results:**

Twenty‐eight controls (27.3 ± 4.1 weeks GA) and 26 CDH (28.1 ± 4.2 weeks GA) fetuses were included. Fractional anisotropy (FA) values were significantly higher (*p* < 0.05) in CDH fetuses relative to controls in 12 of 50 white matter (WM) regions examined based on ANCOVA controlling for GA. After controlling for GA, sex, and CDH side, FA values in the CDH fetuses had a significant positive correlation with observed‐to‐expected lung volumes in 20 of 50 WM regions and with percent predicted lung volumes in 29 of 50 WM regions.

**Conclusion:**

Our data demonstrate evidence of microstructural differences based on DTI indices in the brain between CDH fetuses and age‐matched controls along with correlation with the degree of pulmonary hypoplasia.

**Summary**

- What is already known about this topic:
  ◦ CDH is associated with increased incidence of perinatal brain injury and neurodevelopmental impairment.
  ◦ DTI has become feasible for evaluating white matter microstructure in the fetus in vivo.
- What does this study add:
  ◦ Our data demonstrate initial evidence of microstructural differences based on DTI indices in the brain between CDH fetuses and age‐matched controls along with correlation with the degree of pulmonary hypoplasia.

## 1 Introduction

Congenital diaphragmatic hernia (CDH) is a fetal anomaly associated with high morbidity and mortality with an incidence of 1 in 2000 live births [1]. Mortality has been reported as high as 60% or greater in this population, with lung function representing the most significant source of morbidity in survivors [2]. CDH infants are at high risk of neonatal hypoxic ischemic brain injury and CDH survivors requiring extracorporeal membrane oxygenation (ECMO) are at high risk for long‐term neurologic sequelae including cerebral palsy, sensorineural hearing loss, seizure disorders, cognitive delay and delayed motor skills [3]. However, it is still unclear if there are underlying prenatal neural mechanisms contributing to these poor outcomes. There is existing literature describing global structural abnormalities in the neonatal brain, including thinning of the corpus callosum, enlargement of the subarachnoid fluid spaces, gyral simplification and hypomyelination [4, 5, 6]. Some studies have reported delayed brain development based on in depth sulcal analysis in CDH fetuses, suggesting that brain abnormalities begin in utero.

Fetal MRI, which has become an accessible tool for the clinical evaluation of the developing brain, has the potential to answer some of these questions [7]. Several studies have demonstrated enlarged subarachnoid fluid spaces, decreased cerebral volume and simplification of the gyral sulcal pattern in fetuses with CDH [8, 9, 10, 11]. Diffusion tensor imaging (DTI), a technique sensitive to white matter (WM) abnormalities, has been extensively used in the neonatal brain as a biomarker of various fetal pathologies. Fetal DTI has historically been challenging due to artifacts from excessive fetal motion. With recent advances in imaging data processing and analysis including slice‐to‐volume registration (SVR), fetal DTI has become more robust and reliable, making it possible to extend the study of WM microstructure into the prenatal period [12]. This study aims to assess WM integrity based on in utero DTI in fetuses with CDH.

## 2 Materials and Methods

### 2.1 Study Design and Patients

**FIGURE 1:**
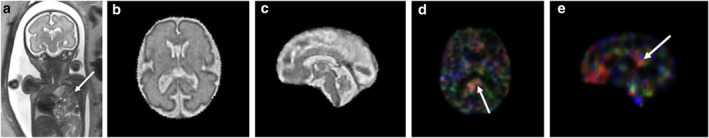
(a–e) Coronal T2‐SSFSE image of a 29 weeks 1 day gestational age fetus (a) showing example of a left congenital diaphragmatic hernia with bowel herniated into the left hemithorax (arrow). Axial (b) and sagittal (c) 3DT2‐SSFSE SVR reformats of the fetal brain in the same infant for anatomic localization. Axial (d) and sagittal (e) color FA maps from DTI of the fetal brain with an arrow pointing to the red fibers of the splenium of the corpus callosum.

This study was a single‐center retrospective review. A clinical database of fetal MRIs performed at Cincinnati Children's Hospital with DTI data sets from January 2020 to April 2021 was compiled and sorted into cohorts of interest. Fetuses with CDH were included as the cohort of interest (Figure 1a). Fetuses with lung pathologies other than CDH and normal fetal brain imaging were selected for the control group. Various clinical parameters were extracted from the fetal MRI radiology reports, including the side of the CDH, observed‐to‐expected lung volumes (O/E) and percent predicted lung volumes (PPLV). A chart review was performed to obtain additional relevant clinical data. This study was compliant with the Health Insurance Portability and Accountability Act and approved by the Institutional Review Board. The requirement for informed consent was waived.

### 2.2 MRI Acquisition

All included fetuses DTI scans were acquired on one of two clinical 1.5 T Philips Ingenia MRI scanners (Philips Healthcare, Best, the Netherlands) scanned with a phased‐array abdominal imaging coil. No sedation was used. Patients were placed in the left lateral decubitus position unless they reported feeling more comfortable in the supine position. Examinations included, per routine clinical protocol, T2 SSFSE (*S*ingle‐*S*hot *F*ast *S*pin *E*cho) images of the fetal brain in the axial, sagittal, and coronal planes with 3 mm (≤ 24 weeks gestational age) or 4 mm (> 24 weeks gestational age) thick interleaved contiguous slices. Fetal DTI data were acquired from the brain in 15 noncollinear diffusion directions with a b‐value of 600 s/mm^2^ and in‐plane resolution of 1.8 mm × 1.8 mm and 4 mm slice thickness.

### 2.3 MRI Data Processing and Analysis

Orthogonally acquired 2D stacks of T2 SSFSE images were motion‐corrected and reconstructed into a single volumetric image with 1 mm^3^ isotropic resolution (Figure 1b,c) using the NiftyMIC toolkit deployed in a Docker image (https://hub.docker.com/r/renbem/niftymic), which additionally calculated an affine‐registration matrix to a gestationally age‐matched template [13]. Fifty WM regions of interest (ROI) were derived from the Johns Hopkins University WM atlas using the Functional MRI of the Brain (FMRIB) Software Library (FSL, version 6.0.4) package's (www.fmrib.ox.ac.uk/fsl) *fnirt* function for non‐linear registration into each gestational age‐matched template space [14, 15]. By applying the inverse affine‐registration matrix, we obtained for each participant 50 WM ROIs aligned to their reconstructed T2 HASTE image.

DTI processing was performed using FSL; diffusion‐weighted images were motion and eddy current correct using FSL's *eddy* routine with intra‐volume subject motion correction enabled to correct for excessive head motion and artifact in fetal DTI [16]. The intra‐volume subject motion correction routine models slice‐to‐volume motion in the diffusion‐weighted images and replaces voxels with missing coverage using a Gaussian prediction process. After preprocessing, the fractional anisotropy (FA) values derived from the diffusion tensor model were calculated (see Figure 1, e.g., of a CDH fetus's T2 SSFSE images [Figure 1b,c] and color‐coded FA map [Figure 1d,e]). Rigid‐body transformation matrices describing the co‐registration of the *b* = 0 diffusion image to the reconstructed T2 SSFSE image were calculated using FSL's *flirt* such that FA values could be extracted from the aforementioned subject‐space WM ROIs Supplemental Material (Supporting Information S1: Figure S1). ROI volumes varied substantially by region, ranging from ∼0.05 to 1.88 cm^3^ with an average volume of 0.4 cm^3^; there were no group differences in volume for any ROI (Supporting Information S1: Figures S2 and S3, Table S1). Participants were excluded from analysis for insufficient image quality as determined by visual inspection; two reviewers (W.Y., J.A.D.) were blinded to participant group status, independently examined color‐coded FA maps and scored each exam as a pass or fail based on subject interpretation of evident anatomical structure and signal to noise. Participants were excluded if either reviewer scored the exam as failed [14, 15].

### 2.4 Statistical Analysis

All statistical analyses were performed using the Statistical Package for Social Sciences (SPSS; IBM). Group differences in FA in each atlas region were assessed using a general linear model framework with CDH as a categorical predictor variable, GA as a continuous predictor variable (mean centered), and the DTI metric as the dependent variables. Partial correlation analysis was performed to test the association between FA and fetal clinical findings (including O/E, PPLV, and postnatal ECMO status). Due to the limited sample size and moderate group difference expected, no multiple comparison correction was used in the analysis of this preliminary study.

## 3 Results

### 3.1 Description of Cohort

After excluding 26 patients for insufficient image quality, a total of 58 patients, including 31 control patients and 27 CDH patients, were identified to have fetal MRI with DTI scans of adequate image quality for analysis. Among these patients, we excluded three patients in the control group (two for spontaneous fetal demise, one for twin pregnancy) and one patient from the CDH group (for termination of pregnancy). In the remaining participants, 4 controls and 10 CDH patients had 2 fetal MRIs; however, the DTI data used in the final analysis were from the first scan. Therefore, a total of 54 fetal MRIs in 28 control patients (27.3 ± 4.1 weeks gestational age) and 26 in CDH patients (28.1 ± 4.2 weeks gestational age) were included. These and other demographic characteristics are provided in Table 1. Lung pathologies observed in the control group included 46.4% (13/28) with a lung mass (presumed congenital pulmonary airway malformation, bronchopulmonary sequestration or hybrid lesion), 46.4% (13/28) with pleural effusions (unilateral or bilateral), 3.6% (1/28) with congenital high airway obstruction (CHAOS) and 3.6% (1/28) with pulmonary hypoplasia in the setting of bladder outlet obstruction. Brain anomalies were described in 26.9% (7/26) of CDH fetal MRIs and included 7.7% (2/26) with mild ventriculomegaly, 3.8% (1/26) with enlarged subarachnoid fluid spaces, 3.8% (1/26) with subependymal cyst with mild ventriculomegaly, 3.8% (1/26) with isolated vermian hypoplasia, 3.8% (1/26) with small supratentorial brain and vermian hypoplasia and 3.8% (1/26) with small supratentorial brain, ventriculomegaly and suspected polymicrogyria. Other fetal MRI findings in the CDH fetuses are summarized in Table 2.

**TABLE 1:** Demographic information.

	CDH MRIs (*n* = 26)	Control MRIs (*n* = 28)	*p*‐value
Maternal age at MRI (years)	28.7 ± 5.4	28.5 ± 6.6	0.87
Gestational age at MRI (weeks)	28.1 ± 4.2	27.3 ± 4.1	0.48
Sex (% female)	57.7% (15/26)	5% (14/28)	0.69
Gestational age at birth (weeks)	37.2 ± 1.5 (*n* = 25)	37.4 ± 2.1 (*n* = 25)	0.70
Birthweight (kg)	2.9 ± 0.6 (*n* = 22)	3.1 ± 0.6 (*n* = 21)	0.23
APGAR score 1 min	3.0 ± 2.7 (*n* = 19)	7.2 ± 1.8 (*n* = 19)	< 0.001
APGAR score 5 min	4.3 ± 2.7 (*n* = 19)	7.9 ± 2.3 (*n* = 19)	< 0.001

**TABLE 2:** CDH information.

	CDH fetal MRIs (*n* = 26)
Observed to expected lung volume ratio (%)	45.5 ± 16.0
Percent predicted lung volume (%)	26.0 ± 15.6
Fetal brain anomalies	26.9% (7/26)
CDH side	73.1% (19/26) left, 26.9% (7/26) right
Liver position	65.4% (17/26) up, 34.6% (9/26) down
Stomach position	65.4% (17/26) up, 34.6% (9/26) down
Neonatal demise	26.9% (7/26)
ECMO	38.5% (10/26)
Trach/g‐tube at discharge	11.5% (3/26)
Brain MRI abnormalities (*n* = 7)	57.1% (4/7)

### 3.2 Fetal DTI Findings

**FIGURE 2:**
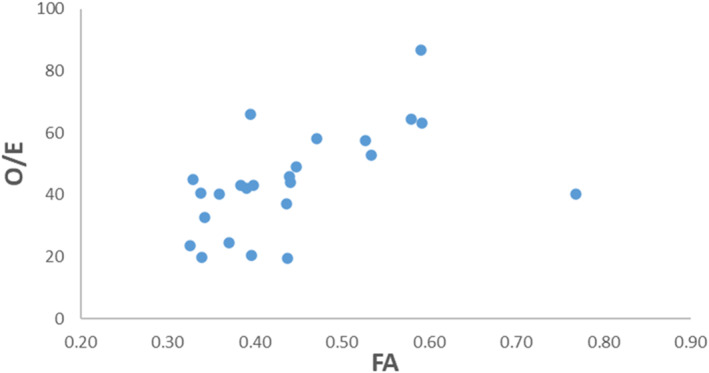
Scatter plot of the association between O/E and FA in genu of corpus callosum controlled for gestational age, fetal sex and CHD side. pCorr = 0.666, df = 14, *p* = 0.005.

When comparing CDH fetuses to controls, FA values in the CDH fetuses were significantly higher in 12 of 50 WM ROIs when controlling for GA (*p* < 0.05, controlled for GA), see Table 3 for details. In the CDH fetuses, there was a significant positive correlation between FA values and O/E in 20 of 50 WM ROIs (all *p* < 0.05) when controlling for GA, fetal sex and CDH side. Figure 2 shows the positive correlation between FA in the genu of the corpus callosum and the O/E ratio. A significant positive correlation was found between FA values and PPLV in 29 of 50 WM ROIs when controlling for GA, sex and CDH side. See Table 4 for details. No significant correlation was found between FA values and postnatal ECMO status.

**TABLE 3:** WM regions with significant group differences in FA values between CDH and control patients.

ROI	CDH	Control	*p*
Body of corpus callosum	0.463 ± 0.124	0.405 ± 0.069	0.041
Superior cerebellar peduncle‐R	0.459 ± 0.150	0.382 ± 0.097	0.032
Cerebral peduncle‐R	0.478 ± 0.148	0.404 ± 0.086	0.036
Superior corona radiata‐R	0.452 ± 0.151	0.389 ± 0.071	0.048
External capsule‐R	0.445 ± 0.136	0.382 ± 0.079	0.035
External capsule‐L	0.432 ± 0.119	0.373 ± 0.074	0.028
Cingulum cingulate gyrus‐L	0.457 ± 0.125	0.400 ± 0.067	0.036
Cingulum hippocampus‐R	0.456 ± 0.130	0.396 ± 0.087	0.050
Superior longitudinal fasciculus‐R	0.443 ± 0.136	0.380 ± 0.086	0.036
Superior fronto‐occipital fasciculus‐R	0.445 ± 0.133	0.384 ± 0.076	0.041
Inferior fronto‐occipital fasciculus‐R	0.455 ± 0.134	0.387 ± 0.099	0.037
Inferior fronto‐occipital fasciculus‐L	0.436 ± 0.135	0.369 ± 0.072	0.023

*Note:* Statistical analysis was performed based on ANCOVA, controlled for GA.

**TABLE 4:** WM regions with significant partial correction between FA and O/E (left) and between FA and PPLV (right) in CDH patients.

	O/E			PPLV		
	rau	df	*p*	rau	df	*p*
gCC	0.666	14	0.005	0.8	14	< 0.001
bCC	0.53	14	0.035	0.577	14	0.019
sCC			ns	0.559	14	0.024
Fornix			ns	0.56	14	0.024
CST_R	0.552	14	0.027	0.503	14	0.047
SCP_R	0.500	14	0.049	0.554	14	0.026
SCP_L	0.595	14	0.015	0.544	14	0.029
CP_R	0.584	14	0.017	0.559	14	0.025
CP_L	0.581	14	0.018	0.692	14	0.003
ALIC_R	0.671	14	0.004	0.699	14	0.003
ALIC_L	0.673	14	0.004	0.77	14	< 0.001
PLIC_R	0.616	14	0.011	0.673	14	0.004
PLIC_L	0.522	14	0.038	0.701	14	0.002
Retrolenticular part of internal capsule_R			ns	0.582	14	0.018
Retrolenticular part of internal capsule_L			ns	0.526	14	0.036
ACR_R			ns	0.505	14	0.046
ACR_L			ns	0.598	14	0.014
SCR_R			Ns	0.509	14	0.044
SCR_L	0.545	14	0.029	0.665	14	0.005
PCR_R			Ns	0.516	14	0.041
PCR_L			ns	0.508	14	0.045
EC_L	0.569	14	0.021	0.746	14	< 0.001
Cingulum cingulategyrus_L	0.555	14	0.026	0.613	14	0.012
Cingulum hippocampus_L	0.549	14	0.028	0.679	14	0.004
Fornix stria terminalis__R	0.507	14	0.045	0.604	14	0.013
Fornix stria terminalis__L			ns	0.522	14	0.038
SLF_R				0.503	14	0.047
SLF_L			ns	0.561	14	0.024
SFOF_R	0.547	14	0.028	0.613	14	0.012
SFOF__L	0.583	14	0.018	0.746	14	< 0.001
IFOF_R	0.556	14	0.025	0.513	14	0.042
IFOF_L	0.505	14	0.046	0.621	14	0.01
Tapetum_L			ns	0.523	14	0.038

*Note:* Statistical analysis was performed based on ANCOVA, controlled for GA, sex, and CDH side.

Abbreviations: ACR, anterior corona radiata; ALIC, anterior limb of internal capsule; bCC, body of corpus callosum; CP, cerebral eduncle; CST, cortico‐spinal cortex; EC, external capsule; gCC, genu of corpus callosum; IFOF, inferior fronto‐occipital asciculus; PCR, posterior corona radiata; PLIC, posterior limb of internal capsule; sCC, splenium of corpus callosum; SCP, superior cerebral peduncle; SCR, superior corona radiata; SFOF, superior fronto‐occipital fasciculus; SLF, superior longitudinal fasciculus.

## 4 Discussion

In this study, we observed significantly higher FA values in the CDH fetuses compared with controls in 12 WM regions of interest including the cerebrum and brainstem after adjusting for gestational age. We also observed a significant positive correlation between FA values and O/E ratios in 20 WM regions of interest and a significant positive correlation with PPLV in 29 WM regions of interest in the CDH fetuses.

The role of fetal MRI in the evaluation of CDH has been well described in lung volume and liver position assessment, both of which have been shown to be predictive of the need for ECMO and neonatal survival [17, 18]. To account for gestational age and variations in fetal size, O/E and PPLV have now become a more widely used assessment of lung volumes on fetal imaging [19, 20]. As a result of the widespread use of fetal MRI in the evaluation of CDH, there is now a growing body of literature describing the fetal brain in this patient population. Multiple studies have reported lower brain volumes, delayed cerebral maturation, and enlarged extra‐axial spaces on fetal MRI [8, 9, 11, 21, 22]. Findings of delayed cerebral maturation and enlarged extra‐axial spaces have also been described in postnatal brain MRIs in this patient population [21, 23]. Evidence of postnatal brain injury on neonatal MRI, CT and head ultrasounds has also been described in infants with prenatally diagnosed CDH [6, 24, 25].

To the best of our knowledge, there is no literature describing DTI findings, neither prenatal nor postnatal, in patients with CDH to date. DTI in the fetus is feasible with the use of slice‐to‐volume registration techniques to compensate for fetal motion and there are now detailed spatiotemporal DTI atlases of the normal fetal brain published [14, 26]. Given that these are relatively recent developments, the use of DTI to evaluate white matter microstructure in the setting of various fetal pathologies is relatively lacking though has been explored in certain conditions. One study observed higher FA values in multiple white matter regions of interest in fetuses with in utero opioid exposure [27]. Additionally, elevated FA values in multiple regions of interest were also observed in a subset of fetuses with Chiari II malformation with the appearance of cerebral edema, with the presence of edema thought to represent a more severe phenotype [28].

Our observation of a positive correlation between FA values and PPLV and O/E ratios is of uncertain etiology. Given that previous work examining the relationship between DTI and histology described lower FA values in association with damage to myelin sheath and axonal membrane, it is possible that fetuses with higher lung volumes have less brain injury and thus higher FA values [29]. Increased postnatal brain injury has been observed in CDH infants with greater degrees of lung hypoplasia on fetal MRI, though it is unclear if these results would translate to the fetal brain [10]. Our study also demonstrates elevated FA values in the CDH fetuses when compared to controls, though the clinical implication of this is unclear. A link between abnormal cerebrovascular hemodynamics has been postulated as a potential cause of increased intracranial fluid in fetuses with CDH, as mass effect on the heart and mediastinal vasculature from the herniated abdominal contents causes impaired venous return, and as a result impaired venous drainage and CSF resorption in the developing brain [10, 30]. Higher FA values can be attributed to extracellular space compression, cytotoxic edema, or inflammation, which would potentially explain our findings [31]. It should be noted that no consensus has been established regarding the normal FA range and the trajectory in association with age in the fetal brain. The rapid development and the change in WM organization and the vulnerability to disturbance during different developmental stages may all affect and complicate the interpretation of our results. It is possible that this may be a trend unique to fetal brain pathology. Within the scope of this study, it is difficult to speculate whether there exist any underlying compensatory mechanisms that alter the trajectory of white matter development. It is uncertain whether there are any specific characteristics that differ in the two groups, such as socioeconomic status, support systems, or other factors, or even the potential interaction effect that were not accounted for in the present study. A greater number of prospective studies with a larger number of patients will be needed to better understand our preliminary findings.

In the present study, as shown in Tables 3 and 4, a number of regions were found to present significant group differences or correlations with clinical outcomes in CDH (O/E and PPLV). There are a wide range of functions that may be affected by these different regions. For example, the genu of the corpus callosum connects the two hemispheres for information integration and contributes to neurocognition and motor functions. The cingulum and cingulate gyrus are critical for emotion, behavior, and memory processing. Inferior fronto‐occipital fasciculus contributes to language, semantics, inhibition and the control of action. Within the scope of this study, it is difficult to provide a systemic interpretation and speculate the underlying mechanism (potentially hemodynamic) on how CDH may affect all these various functional domains during the development.

Our study has some limitations. For one, its retrospective nature limits its internal validity. Second, its single institutional nature involving a relatively small number of patients limits its external validity. The statistical significance in the results was moderate and did not go through multiple comparison corrections. Therefore, our findings need to be validated with a large scale prospective study to allow for rigorous statistical analysis. On the other hand, it should be noted that in a true null case, the distribution of *p*‐values should be uniform between 0 and 1. In contrast, we see a heavily right‐skewed distribution of *p*‐values in our data where 12, 20, 29 out of 50 ROIs presented *p*‐values below 0.05 in the group comparisons and the correlation analysis with O/E and PPLV, respectively. Therefore, we believe that we are detecting real signals and not reporting purely spurious results. Another limitation is the excessive head motion artifact, which is common in fetal imaging and one of the main challenges faced in fetal DTI performance. Despite using SVR to improve image quality, 31% (26/84) of patients had to be excluded for excessive fetal motion, which in our experience was relatively robust given these were observed retrospectively in the clinical setting. Super‐resolution reconstruction methods have been shown to improve the image and data quality by scanning in multiple orthogonal planes and show promise for future studies though they come at the cost of increased imaging time [32]. Lack of more robust clinical outcomes in our CDH population is also a limitation in the interpretation of our findings; however, these are challenging to obtain in this population given the high rate of mortality and confounding co‐morbidities. Finally, the lack of a prospectively recruited matched normal control group also limits the validity of our findings. We chose to use age matched patients with suspected lung lesions and a reportedly normal clinical brain MRI as our control group because of the lack of literature describing an association with brain anomalies and the potential of reducing inherent lung pathology as a confounding variable. We acknowledge that this selected control group has some limitations, including the varying degrees of altered pulmonary circulation encountered in fetuses with lung masses; however, this was weighed against the limitations of other fetal pathologies commonly referred for fetal MRI in our center. There are many published studies describing normal reference ranges for fetal brain biometric measurements using fetuses with reported extra‐CNS abnormalities because fetal MRI is not routinely performed in clinical practice in normal fetuses; however, we acknowledge that this has some limitations and a prospective study in a larger number of patients would be more informative [33].

In conclusion, this study demonstrates significant DTI abnormalities in multiple white matter regions of interest in fetuses with CDH and a strong correlation between DTI parameters and lung volume indices. We hypothesize that this is related to altered hemodynamics secondary to mass effect from the hernia contents on the heart and other mediastinal vascular structures resulting in increased intracranial venous pressure, and as a result disrupting normal white matter development. Larger prospective studies will be needed to demonstrate the true value of these findings in the context of prenatal counseling, prenatal intervention and perinatal management in order to create treatments that will optimize long‐term outcomes.

## Ethics Statement

This study was approved by the institutional review board (IRB#2021‐0141).

## Consent

The authors have nothing to report.

## Conflicts of Interest

The authors declare no conflicts of interest.

## Supporting information

Supporting Information S1

## Data Availability

The data that support the findings of this study are available from the corresponding author upon reasonable request.
