# Supplementary material for: Diffusion Tensor Imaging of Fetuses With Congenital Diaphragmatic Hernia
Source: Prenat Diagn. 2025 Jun 11;45(8):1045–52. doi: 10.1002/pd.6835 (PMC12254437; doi:10.1002/pd.6835)
Supplement: Supplementary file 1 — Supporting Information S1 [file PD-45-1045-s001.docx]

**Supplemental Material**


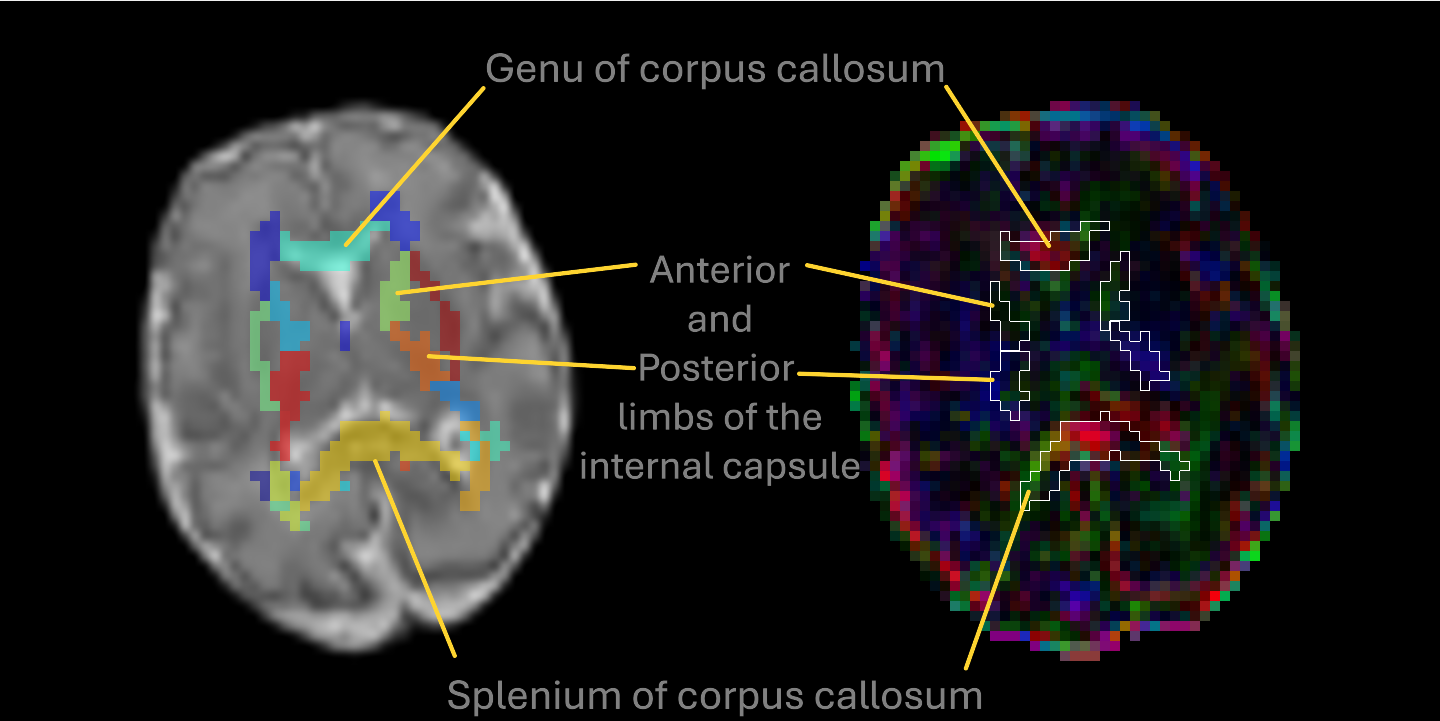


**Supplemental Figure S1**: At left, white matter ROIs overlaid on the subject-space super-resolution reconstructed T2-weighted SSFSE image. At right, the perimeter of six of those ROIs overlaid on the subject-space color-coded FA map (note: only six are shown for clarity – additional and smaller ROIs make the boundaries difficult to distinguish).


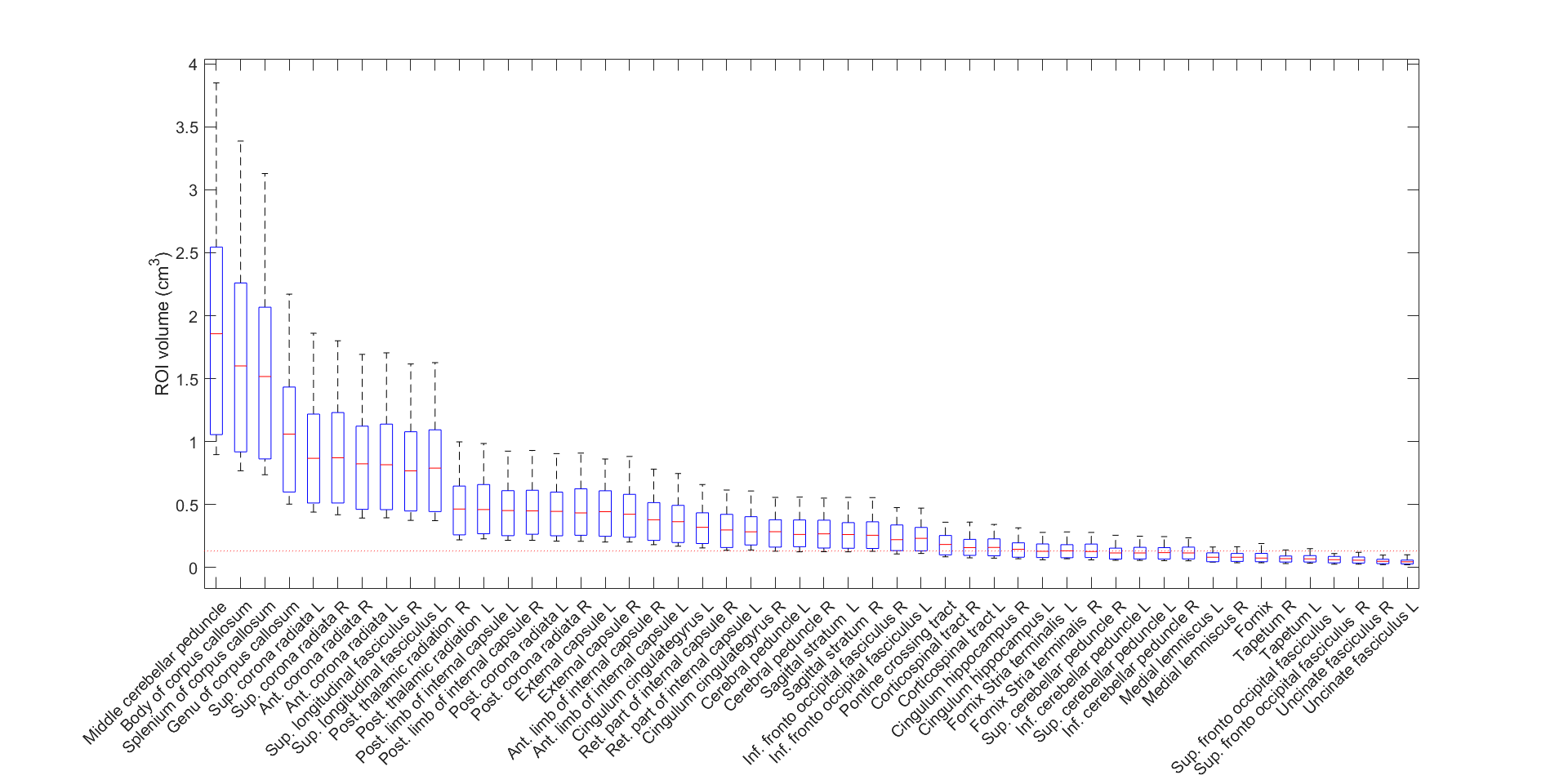


**Supplemental Figure S2:** Distributions of subject-space volumes for each ROI. Red lines represent median volumes, blue boxes indicate interquartile range, black lines extend to minimum and maximum observed volumes. The dashed red line represents the volume equivalent to 10 voxels at the resolution of the diffusion weighted imaging data (1.8 x 1.8 x 4 mm)


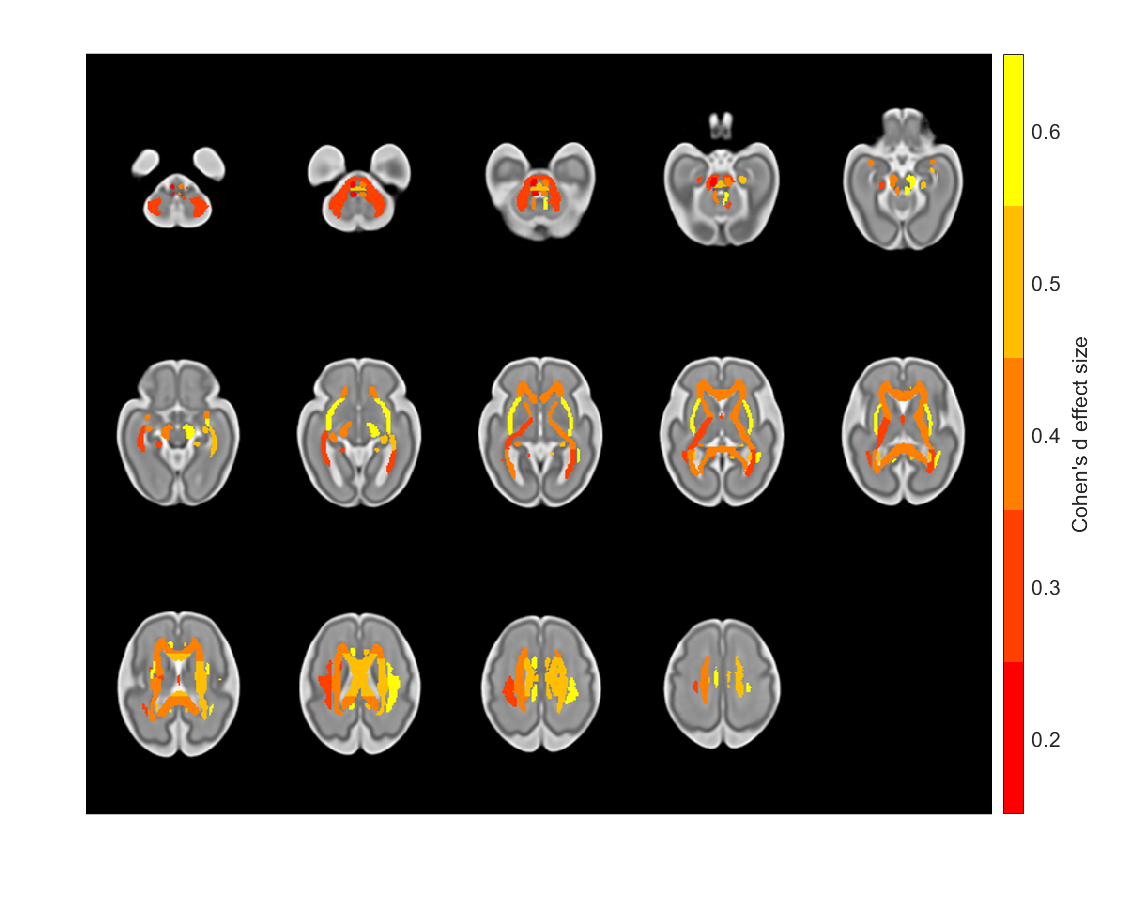


**Supplemental Figure S3**: The Cohen’s d effect size for all 50 white matter ROIs, overlaid on the 28-week T2-weighted fetal template. CDH patients exhibited higher mean FA values compared to controls in all 50 ROIs. Effects tended to be somewhat stronger in the right-hemisphere.

**Supplemental Table S1**: The mean and standard deviation (in cm^3^) of ROI volumes, split by group status, and the results of the associated two-sample t-tests. There was no significant group difference in volume for any ROI.

|  | CDH | | Controls | |  |  |
| --- | --- | --- | --- | --- | --- | --- |
| ROI | mean | std | mean | std | T-stat | p-value |
| Middle cerebellar peduncle | 1.97 | 0.86 | 1.79 | 0.73 | 0.79 (52) | 0.433 |
| Pontine crossing tract | 0.19 | 0.09 | 0.17 | 0.07 | 0.77 (52) | 0.443 |
| Genu of corpus callosum | 1.11 | 0.48 | 1.01 | 0.41 | 0.80 (52) | 0.425 |
| Body of corpus callosum | 1.73 | 0.77 | 1.57 | 0.65 | 0.80 (52) | 0.428 |
| Splenium of corpus callosum | 1.59 | 0.70 | 1.46 | 0.59 | 0.75 (52) | 0.455 |
| Fornix | 0.08 | 0.04 | 0.07 | 0.03 | 0.88 (52) | 0.383 |
| Corticospinal tract R | 0.17 | 0.08 | 0.16 | 0.06 | 0.81 (52) | 0.421 |
| Corticospinal tract L | 0.17 | 0.08 | 0.16 | 0.07 | 0.74 (52) | 0.461 |
| Medial lemniscus R | 0.09 | 0.04 | 0.08 | 0.03 | 0.82 (52) | 0.414 |
| Medial lemniscus L | 0.09 | 0.04 | 0.08 | 0.04 | 0.74 (52) | 0.463 |
| Inf. cerebellar peduncle R | 0.12 | 0.05 | 0.11 | 0.05 | 0.87 (52) | 0.386 |
| Inf. cerebellar peduncle L | 0.12 | 0.05 | 0.11 | 0.04 | 0.84 (52) | 0.403 |
| Sup. cerebellar peduncle R | 0.12 | 0.06 | 0.11 | 0.05 | 0.61 (52) | 0.542 |
| Sup. cerebellar peduncle L | 0.12 | 0.06 | 0.11 | 0.05 | 0.75 (52) | 0.459 |
| Cerebral peduncle R | 0.28 | 0.13 | 0.26 | 0.11 | 0.79 (52) | 0.436 |
| Cerebral peduncle L | 0.29 | 0.13 | 0.26 | 0.11 | 0.89 (52) | 0.379 |
| Ant. limb of internal capsule R | 0.39 | 0.17 | 0.36 | 0.15 | 0.82 (52) | 0.418 |
| Ant. limb of internal capsule L | 0.38 | 0.17 | 0.34 | 0.14 | 0.77 (52) | 0.443 |
| Post. limb of internal capsule R | 0.47 | 0.20 | 0.43 | 0.17 | 0.86 (52) | 0.395 |
| Post. limb of internal capsule L | 0.47 | 0.21 | 0.43 | 0.17 | 0.77 (52) | 0.445 |
| Retro. part of internal capsule R | 0.31 | 0.14 | 0.29 | 0.12 | 0.74 (52) | 0.461 |
| Retro. part of internal capsule L | 0.31 | 0.14 | 0.28 | 0.12 | 0.78 (52) | 0.439 |
| Ant. corona radiata R | 0.86 | 0.38 | 0.79 | 0.32 | 0.79 (52) | 0.435 |
| Ant. corona radiata L | 0.86 | 0.39 | 0.78 | 0.33 | 0.77 (52) | 0.446 |
| Sup. corona radiata R | 0.93 | 0.42 | 0.85 | 0.35 | 0.78 (52) | 0.436 |
| Sup. corona radiata L | 0.94 | 0.41 | 0.85 | 0.34 | 0.85 (52) | 0.402 |
| Post. corona radiata R | 0.46 | 0.21 | 0.42 | 0.18 | 0.83 (52) | 0.412 |
| Post. corona radiata L | 0.46 | 0.20 | 0.42 | 0.17 | 0.75 (52) | 0.455 |
| Post. thalamic radiation R | 0.50 | 0.22 | 0.46 | 0.19 | 0.76 (52) | 0.450 |
| Post. thalamic radiation L | 0.49 | 0.22 | 0.45 | 0.19 | 0.76 (52) | 0.450 |
| Sagittal stratum R | 0.27 | 0.12 | 0.25 | 0.10 | 0.80 (52) | 0.428 |
| Sagittal stratum L | 0.28 | 0.12 | 0.26 | 0.10 | 0.70 (52) | 0.485 |
| External capsule R | 0.45 | 0.20 | 0.41 | 0.17 | 0.79 (52) | 0.435 |
| External capsule L | 0.46 | 0.20 | 0.42 | 0.17 | 0.75 (52) | 0.457 |
| Cingulum cingulategyrus R | 0.30 | 0.13 | 0.27 | 0.11 | 0.82 (52) | 0.414 |
| Cingulum cingulategyrus L | 0.34 | 0.15 | 0.31 | 0.12 | 0.73 (52) | 0.471 |
| Cingulum hippocampus R | 0.15 | 0.07 | 0.14 | 0.06 | 0.82 (52) | 0.417 |
| Cingulum hippocampus L | 0.14 | 0.06 | 0.13 | 0.05 | 0.88 (52) | 0.385 |
| Fornix Stria terminalis R | 0.14 | 0.06 | 0.13 | 0.05 | 0.75 (52) | 0.459 |
| Fornix Stria terminalis L | 0.14 | 0.06 | 0.13 | 0.05 | 0.74 (52) | 0.465 |
| Sup. longitudinal fasciculus R | 0.83 | 0.36 | 0.75 | 0.31 | 0.81 (52) | 0.424 |
| Sup. longitudinal fasciculus L | 0.82 | 0.36 | 0.75 | 0.31 | 0.79 (52) | 0.432 |
| Sup. fronto occipital fasciculus R | 0.06 | 0.03 | 0.06 | 0.02 | 0.67 (52) | 0.505 |
| Sup. fronto occipital fasciculus L | 0.06 | 0.03 | 0.06 | 0.02 | 0.81 (52) | 0.424 |
| Inf. fronto occipital fasciculus R | 0.25 | 0.11 | 0.22 | 0.09 | 0.85 (52) | 0.399 |
| Inf. fronto occipital fasciculus L | 0.25 | 0.10 | 0.22 | 0.09 | 0.90 (52) | 0.374 |
| Uncinate fasciculus R | 0.05 | 0.02 | 0.04 | 0.02 | 0.75 (52) | 0.457 |
| Uncinate fasciculus L | 0.05 | 0.02 | 0.04 | 0.02 | 0.74 (52) | 0.460 |
| Tapetum R | 0.07 | 0.03 | 0.07 | 0.03 | 0.70 (52) | 0.486 |
| Tapetum L | 0.07 | 0.03 | 0.07 | 0.03 | 0.73 (52) | 0.467 |
